# Skeleton-Based Emotion Recognition Based on Two-Stream Self-Attention Enhanced Spatial-Temporal Graph Convolutional Network

**DOI:** 10.3390/s21010205

**Published:** 2020-12-30

**Authors:** Jiaqi Shi, Chaoran Liu, Carlos Toshinori Ishi, Hiroshi Ishiguro

**Affiliations:** 1 Graduate School of Engineering Science, Osaka University, Osaka 565-0871, Japan; ishiguro@irl.sys.es.osaka-u.ac.jp; 2 Advanced Telecommunications Research Institute International, Kyoto 619-0237, Japan; chaoran.liu@atr.jp (C.L.); carlos@atr.jp (C.T.I.); 3 Interactive Robot Research Team, Robotics Project, RIKEN, Kyoto 351-0198, Japan

**Keywords:** emotion recognition, gesture, skeleton, graph convolutional networks, self-attention

## Abstract

Emotion recognition has drawn consistent attention from researchers recently. Although gesture modality plays an important role in expressing emotion, it is seldom considered in the field of emotion recognition. A key reason is the scarcity of labeled data containing 3D skeleton data. Some studies in action recognition have applied graph-based neural networks to explicitly model the spatial connection between joints. However, this method has not been considered in the field of gesture-based emotion recognition, so far. In this work, we applied a pose estimation based method to extract 3D skeleton coordinates for IEMOCAP database. We propose a self-attention enhanced spatial temporal graph convolutional network for skeleton-based emotion recognition, in which the spatial convolutional part models the skeletal structure of the body as a static graph, and the self-attention part dynamically constructs more connections between the joints and provides supplementary information. Our experiment demonstrates that the proposed model significantly outperforms other models and that the features of the extracted skeleton data improve the performance of multimodal emotion recognition.

## 1. Introduction

Multimodal emotion recognition has attracted a lot of attention due to its wide range of application scenarios. Many previous research efforts on emotion recognition process the information from different modalities and use multimodal clues to infer the emotional states. Body gestures are an integral part of nonverbal communication and deliver extremely important supplementary information when expressing emotions [1]. A robot or interactive system with the ability to recognize emotions from body movement can bring significant benefits to many applications, such as biometric security, healthcare, and gaming [2]. Body movement information has good robustness and can be a better alternative for emotion recognition from a distance [3]. However, considerably less work has been done on automatic emotion recognition using body movement.

The dynamic human skeleton is the most intuitional and natural method for depicting human actions, and it does so by structuring the body movement as a sequence of joint positions. The scarcity of labeled data containing 3D skeleton data is one of the significant reasons the body gesture modality has seldom received attention in emotion recognition tasks. Although several multimodal emotional databases include skeleton coordinates, e.g., the multimodal database created by Sapiński et al. (560 samples) [4] and the emoFBVP database (1380 samples) [5], the relatively small amount of samples and the lack of interpersonal dialogue and interaction limit the generalization and robustness of the models trained on them. By contrast, research using speech signals, textual transcriptions and facial expressions mostly evaluate their models on large open-source multimodal emotional benchmark datasets, such as the interactive emotional dyadic motion capture database (IEMOCAP, over 10000 samples) [6]. However, these databases do not contain skeleton data representing the gesture modality, which makes them difficult to use in gesture emotion recognition.

Since deep learning has revolutionized many fields and had an outstanding performance, some recent research has utilized neural networks on gesture-based emotion recognition [7,8,9], which fed video frames or a sequence of joint coordinates into neural networks, e.g., convolutional neural networks (CNNs) and recurrent neural networks (RNNs), to extract emotion-related features and make predictions. However, since the spatial connections and graphic structures between joints are seldom explicitly considered by these methods using image sequences and skeletons, the ability to understand the emotion expressed by the body movement is relatively limited.

In the field of action recognition, the Spatial Temporal Graph Convolutional Network (ST-GCN) [10] abstracts the skeletal structure of the human body as a spatial graph, in which the vertexes are joints and the edges represent natural bone connections, and then constructs a graph network using spatial-temporal convolutional blocks to extract features and make predictions. However, ST-GCN has some shortcomings: the topology of the graph is fixed and does not change with the information of each node, which means that the flow direction of information between nodes is predefined and limited to the natural connection between the joints of the body [11,12]. This is also unfavorable for emotion prediction tasks based on skeletal data and there is a lack of flexible connections that are necessary for emotional expression using body gestures. For example, when people express anger, the joints of the limbs, especially the two hands, tend to have relatively high gestural dynamics characteristics, e.g., movement speed and amount of movement [13]. A strong connection between these joints is likely necessary, but the fixed graph structure does not guarantee that the network can capture the appropriate dependency.

To solve these problems, we make the following contributions: (i) we extract 3D skeleton movement data from raw video based on pose estimation and the method can be used to expand existing databases to alleviate the lack of labeled data. (ii) We propose a self-attention enhanced spatial temporal graph convolutional network for skeleton-based emotion recognition, in which the self-attention part of the spatial graph convolutional layer dynamically constructs connections between the nodes of the entire graph and provides supplementary information for the spatial graph convolution. The model outperforms baselines by a significant margin. (iii) We fuse the representations extracted by the proposed model with the audio and text high-level features, to use audio, text, and skeleton modalities simultaneously. The performance significantly exceeds that of the bimodal model using only audio and text information, which shows the effectiveness of the extracted modality.

## 2. Related Work

### 2.1. Emotion Recognition

Emotion not only plays a crucial role in interaction, decision making, and cognitive processes [14], but also further affects the accuracy of our memory of some events [15]. In human-human interaction, people naturally express and recognize their emotions through multiple modalities including speech, semantics, facial cues, and physical movement [16]. In recent years, the automatic detection of human emotional states has consistently drawn attention from researchers, due to its wide range of applications and growing demand in many different areas such as human-robot interaction, safety driving, website customization, and education [17,18]. For example, a driver emotion detection system can automatically infer the driver’s emotional state and take corresponding measures to ensure road safety and human health [19]. Many previous research efforts on emotion recognition process the information from different modalities and use multimodal clues to infer the emotional states, and they show improvement of the overall performance by multimodal fusion [20]. Tzirakis et al. applied convolutional neural networks to extract features from the speech and the facial expression and utilized long short-term memory networks to model the context and improve the performance [21]. Heusser et al. combined pretrained language models with speech emotion recognition and achieved a better accuracy [22]. However, these works rarely consider gesture as the source of information on recognizing emotions.

### 2.2. Gesture-Based Emotion Recognition

There have been some studies about gesture-based emotion recognition over the past few years [23]. For example, Ahmed et al. [2,24] applied a feature selection algorithm to select features from the movement feature groups and proposed a genetic algorithm to recognize emotions from body movement. A big challenge of emotion recognition is to design suitable features to capture the emotional properties of body movement. Inspired by the great performance improvement brought by deep learning in many important tasks, some researchers also used deep learning in an end-to-end manner for gesture-based emotion recognition. Filntisis et al. [8] fed flattened 2D pose data into deep neural networks to get a representation and fused it with facial features to predict emotion, which achieved better results than the model using only facial data. Karumuri et al. [25] used convolutional neural networks for classification of emotions from (motion capture) data of dance movements. Deng et al. [26] proposed an attention-based bidirectional LSTM to explore the relationship between human behavior data and emotions.

Most of these studies used open-source multimodal datasets or created new datasets without a large amount of data. Since labeled emotional gesture data is scarcer than speech and facial data, these studies have been limited. Moreover, single-person action performance instead of natural dialogue-based interactive data has been used in these studies, which makes learning the natural expression of human emotions in interactive scenes difficult. Besides, most of the existing deep learning methods for gesture emotion recognition are based on RNNs or CNNs, which cannot represent the spatial connections of joints.

### 2.3. Graph Neural Networks

Graph is a data structure composed of a series of nodes and edges. As non-Euclidean data, graph data have no regular spatial structure, and their complexity poses a great challenge to existing machine learning algorithms [27]. Recently, graph neural networks (GNNs) have been used in a wide variety of difficult tasks for previous machine learning algorithms, because of their ability to model the data generated from non-Euclidean domains and capture the internal dependence of the data [28], and they have achieved extensive success [29,30].

Encouraged by the success of CNNs in the computer vision domain, convolutional graph networks (GCNs) that generalize convolutions to graph data have attracted an increasing interest [27]. These approaches can be categorized as spectral approaches and spatial approaches [31]. Spectral approaches define graph convolutions by using the graph Fourier transform. Bruna et al. [32] first developed a spectral-based graph convolution, which is limited because of the huge calculation burden. The approaches proposed by Defferard et al. [33] improved efficiency and solved this problem. Spatial approaches directly use the topology of the graph and apply the convolution filter based on the neighborhood information of the graph. Monti et al. [34] proposed a spatial-domain model and generalized CNN architectures to non-Euclidean domains. Hamilton et al. [35] proposed a general inductive framework that samples and aggregates features from a node’s local neighborhood to generate embeddings. This work follows the spatial approaches.

## 3. Skeletal Data Extraction

We extract dynamic body skeleton data from raw video of a large existing open-source emotional database to add a new modality representing gestures. To do this, we use a human pose estimation method to draw a temporal sequence of joint positions in the form of 3D coordinates. Then some prepossessing steps are applied for the data before feeding it into the emotion recognition model.

### 3.1. Human Pose Estimation

Human pose estimation is used to localize the human joints in images or videos, obtain 2D or 3D coordinates of the joints, and reconstruct the coordinate representations of the human body. With the increase of computing resources and the amount of data, the pose estimation method using learning has gradually matured and had considerable success. We first apply AlphaPose [36,37,38], an accurate open-source pose estimator, to detect the 2D position in the image frames of videos. The AlphaPose system pretrained on the COCO database [39] is used for 2D keypoint detection. Then we project the 2D detected results into 3D coordinates, using a pretrained fully convolutional model based on dilated temporal convolutions proposed by Pavllo et al. [40]. In this way, the dynamic skeleton representations of the human body in form of 3D coordinates are obtained.

### 3.2. Data Preprocessing

In the 2D pose estimation results, there is high-frequency noise because of the image quality and the estimation error, which causes instability of the 3D coordinates. We add a low-pass filter after 2D joint position detection to filter out the noise and obtain clean skeleton data.

Since the lower halves of the actors’ bodies are not visible in the video clips most of the time, the reliability of the estimation results of this part of the body is much lower than that of the upper body. Therefore, the lower body part is removed from the skeleton graph and only 10 joints of the upper body are considered in our research.

## 4. Proposed Networks

### 4.1. Graph Convolutional Network

#### 4.1.1. Skeleton Graph Construction

**Figure 1:**
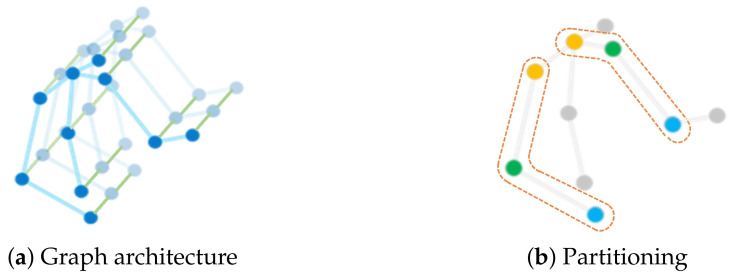
Skeleton graph construction. (**a**) The spatial-temporal graph contains vertexes (blue dots), spatial edges (blue lines), and temporal edges (green lines). (**b**) The dots of different colors in the dashed box represent 3 subsets according to the spatial configuration partitioning strategy, that is, the root node (green), the nodes closer than the root node (yellow), and the nodes farther than the root node from the spinebase joint (blue).

The extracted body skeleton in each frame is represented in the form of 3D coordinate vectors of human joints. The dynamic motion data of each sample is a time sequence with different timesteps because of the variable lengths of the video clips. Following the work of [10], we construct an undirected spatial-temporal graph *G* composed of vertexes *V* and edges *E*, represented as G=(V,E). The spatial-temporal graph is a representation of body movement along the spatial and the temporal dimensions and retains gestural static and dynamics characteristics, e.g., movement speed and amount of movement, which are helpful in recognizing emotions accurately [13,41]. Figure 1a shows the spatial-temporal graph of a skeleton sequence, where the vertexes denote the joints of the upper body, the blue lines represent the spatial edges based on the natural connection of human joints, and the green lines indicate the temporal edges that exist between two consecutive timesteps of the same joints. As illustrated in Figure 1b, the dots of different colors (in the dashed box) represent different subsets of the adjacency matrix of the graph based on the partitioning strategy. The root node and its neighboring nodes are divided into three subsets according to their distance from the body center (spinebase), i.e., the root node, the nodes closer than the root node, and the nodes farther than the root node.

#### 4.1.2. Self-Attention Enhanced Spatial Graph Convolutional Layer

As described in Section 1, in the work of Yan et al. [10], the calculation of graph convolution is based on the fixed topology of the graph, which may not be appropriate for emotion recognition tasks. To solve the problem, not only local neighborhood nodes that are predefined by the natural connection of the human body but also other nodes with high relevance of information need to be considered in the process of message passing. Therefore, we propose a self-attention enhanced spatial graph convolutional layer, in which the self-attention mechanism calculates the weighted sum of the values of all nodes to aggregate features from the entire graph and provide supplementary information for the spatial graph convolution module.

**Figure 2:**
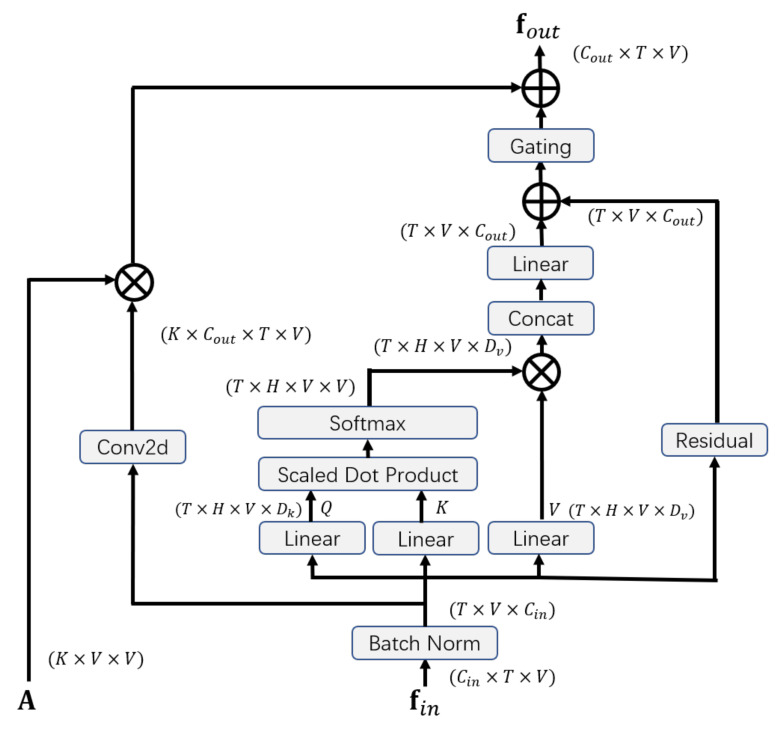
Illustration of self-Attention enhanced spatial graph convolutional layer.

The structure of our self-attention enhanced spatial graph convolutional layer is illustrated in Figure 2. The layer is composed of three parts: the graph convolutional part, the self-attention part, and the gating mechanism.

Graph convolutional part: For the spatial graph convolutional part, the structure of the graph is predefined by the the adjacency matrix A. According to the partitioning strategy, A is divided into *K* subsets, i.e., $A=\sum_{k}^{K}A_{k}$, $A_{k}\in R^{V\times V}$. The input $f_{in}$ is a tensor with the shape $\left(C_{in}\times T\times V\right)$. First, a convolution operation is applied for the input:

(1) $$f=W_{k}f_{in},$$

where $W_{k}\in R^{\left(K\times C_{out}\right)\times C_{in}\times 1\times 1}$ is a trainable matrix of the 1×1 convolution. Similarly, f is also divided into *K* subsets, i.e., $f=(f_{1},\ldots ,f_{K})$, $f_{k}\in R^{C_{out}\times T\times V}$. The output of the spatial graph convolutional part is calculated as:

(2) $$f_{g}=\sum_{k}^{K}f_{k}\left(A_{k}⊗M_{k}\right),$$

where $M_{k}\in R^{V\times V}$ is a trainable weight matrix, and ⊗ denotes the element-wise product between matrices.

Self-attention part: The self-attention part applies a multi-head scaled dot product attention on the graph. In the field of Natural Language Processing, Vaswani et al. proposed multi-head self-attention, in which the queries and the corresponding keys are used to compute the weight and the weighted sum of the values is calculated as the output [42]. The self-attention model could be regarded as a spatial graph neural network with a fully-connected graph, in which the feature vectors of joints are nodes and every pair of nodes is connected. The model aggregates features from every node of the graph instead of neighborhood nodes when updating representations of nodes. First, the input is reshaped as a tensor with the shape $\left(T\times V\times C_{in}\right)$, where *T* is the temporal length, *V* is the number of joints, and $C_{in}$ is the number of input channels.

For each timestep in one sample, we represent the feature vector of each joint as $a_{i}\in R^{C_{in}}$, whose amount is V. The set of the vectors corresponding to the joint representations is $A=\left\{a_{1},\ldots ,a_{V}\right\}$. Then the similarity between the features of query *i* and the features of key *j* is calculated by the scaled dot product for each head:

(3) $$e_{ij}^{head}=\frac{\left(a_{i}W_{Q}^{head}\right){\left(a_{j}W_{K}^{head}\right)}^{T}}{\sqrt{d_{k}}},$$

where $W_{Q}^{head}\in R^{C_{in}\times d_{k}}$ and $W_{K}^{head}\in R^{C_{in}\times d_{k}}$ are learnable matrices for one head, $d_{k}$ is the dimension of query. After this, the softmax function is applied to obtain the attention coefficient α:

(4) $$\alpha_{ij}^{head}=\frac{\exp e_{ij}^{head}}{\sum_{k=1}^{V}\exp e_{ik}^{head}}.$$

The weighted sum of values for each head is $s^{head}=\left(s_{1}^{head},\ldots ,s_{V}^{head}\right)\in R^{V\times d_{v}}$, where $d_{v}$ is the dimension of value. The calculation of $s_{i}^{head}$ is formulated as:

(5) $$s_{i}^{head}=\sum_{j}^{V}\alpha_{ij}^{head}\left(a_{j}W_{V}^{head}\right),$$

where $W_{V}\in R^{C_{in}\times d_{v}}$ is a learnable matrix. Then we calculate the self-attention result $f_{o}$:

(6) $$f_{o}=Concat\left(s^{1},\ldots ,s^{H}\right)W_{O},$$

where *H* is the number of attention heads, and $W_{O}\in R^{\left(H\times d_{v}\right)\times C_{out}}$ is a parameter matrix. To stabilize the training procedure, a residual is added for the output of the self-attention part:

(7) $$f_{a}=f_{o}+Residual\left(f_{in}\right),$$

(8) $$Residual\left(f_{in}\right)=\left\{\begin{array}{ccc} f_{in}W_{R} & \; & C_{in}\neq C_{out}\\ f_{in} & \; & C_{in}=C_{out} \end{array}\right.,$$

where $W_{R}\in R^{C_{in}\times C_{out}}$ is a parameter matrix that transforms the number of input channels to the number of output channels.

Gating mechanism: Considering that the contributions of the two parts may be different for updating representations of nodes, we use a trainable coefficient *r* to adjust the weight of the self-attention part, which is formulated as:

(9) $$f_{out}=\frac{f_{g}+r\times f_{a}}{2},$$

where the trainable coefficient *r* is initialized as 1.

#### 4.1.3. Self-Attention Enhanced Spatial Temporal Graph Convolutional Network

**Figure 3:**
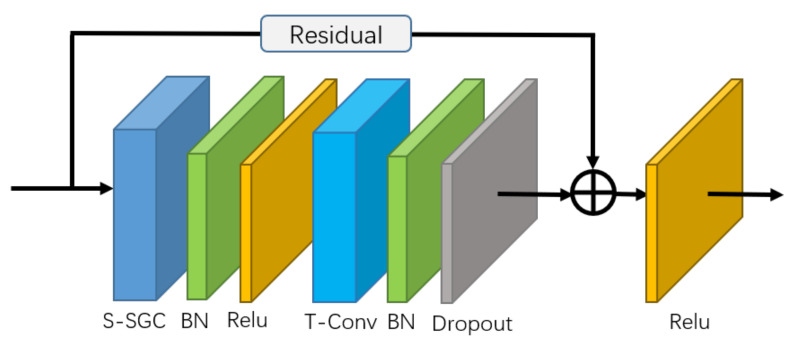
Illustration of basic graph convolutional block. The residual connection is applied to guarantee the stability of training. S-SGC represents the self-attention enhanced spatial graph convolutional layer. T-Conv represents the temporal convolutional layer.

The basic block of the self-attention enhanced spatial temporal graph convolutional network (S-STGCN) is composed of a self-attention enhanced spatial graph convolutional layer, a temporal convolutional layer, and several functional layers (See Figure 3). The self-attention enhanced spatial graph convolutional layer is used to aggregate the information of the joints along the spatial dimension. The temporal convolutional layer of the network is the same as that of the ST-GCN, i.e., apply the convolution with receptive field $\left(K_{t},1\right)$ on the feature vectors of each node along the temporal dimension.

**Figure 4:**
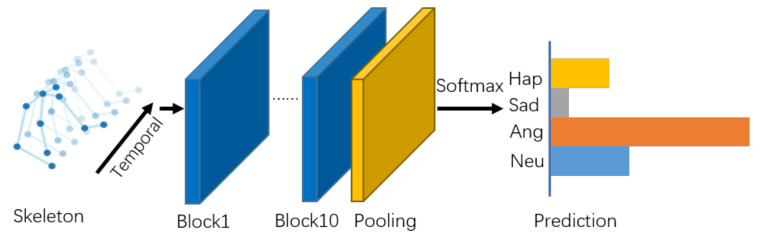
Overview of our proposed self-attention enhanced spatial temporal graph convolutional network.

As illustrated in Figure 4, there are 10 basic blocks in the model, with output channels of 32, 32, 32, 32, 64, 64, 64, 128, 128, and 128. After that, the output tensor is fed into a global average pooling layer to get an emotional feature vector for each sample. Finally, the vectors are passed into the output layer with a softmax function to obtain the prediction of the emotion classes.

#### 4.1.4. Two-Stream Architecture

In some previous works of action recognition [11,43], in addition to the joint positions, the second-order feature, i.e., the bone information representing the lengths and orientations of the human bones, has also been proved to be useful for skeleton-based action recognition tasks. Considering the bone information may also play an important role in the recognition of emotions in a similar way, we construct a two-stream network to simultaneously use the joint information and bone information.

Similar to the work of [11], we define the bone features as the vectors from the source nodes to the target nodes. Each bone is between two adjacent joints, in which the joint close to the center (spinebase) of the skeleton is defined as the source node, and the joint far away from the center is defined as the target node. For example, the bone vector between the source node $v_{1}$ and the target node $v_{2}$ is represented as $e_{1,2}=v_{2}-v_{1}$.

**Figure 5:**
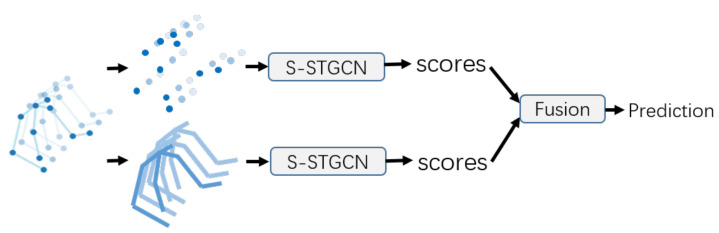
Two-stream network. The two self-attention enhanced spatial temporal graph convolutional networks (S-STGCNs) take the joint information and the bone information as input respectively. The fusion of the outputs of these models are used for final prediction.

The architecture of the two-stream self-attention enhanced spatial temporal graph convolutional network (2s-S-STGCN) is illustrated in Figure 5. The joint data representing the positions of the joints and the bone data representing the lengths and directions of the bones are fed into two S-STGCNs and each stream is trained respectively. Then the output tensors of the two streams are fused to predict emotion labels.

### 4.2. Multimodal Emotion Recognition Network

**Figure 6:**
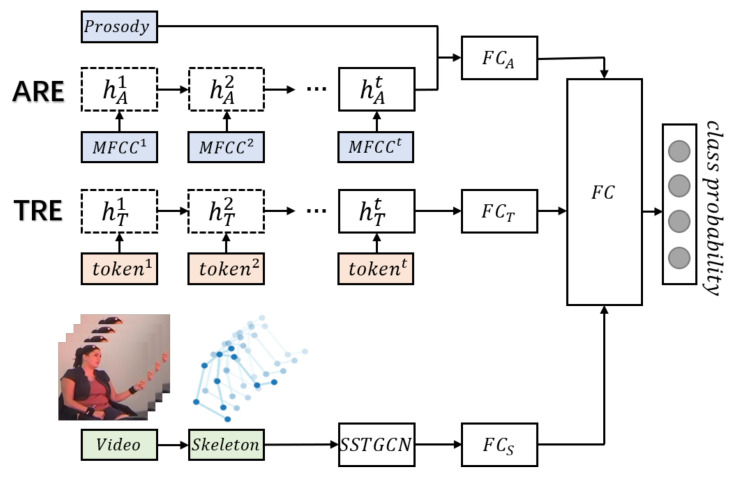
Skeleton enhanced emotion recognition network. Each FC represents a fully connected layer. The three fully connected layers on the left ($FC_{A},FC_{T},FC_{S}$) are used for adjusting the dimensions of the representation vectors of audio, text, and skeleton. The fully connected layer on the right is used to learn the weights of these features and make final prediction.

We construct a skeleton enhanced emotion recognition network (SERN), which integrates text and audio information with the features extracted by the self-attention enhanced spatial temporal graph convolutional network (See Figure 6). The multimodal dual recurrent encoder (MDRE) [44], composed of an audio recurrent encoder (ARE) and a text recurrent encoder (TRE), is used to extract high-level representations of audio and text, which takes as input prosody features, MFCC features and text transcripts. In the ARE, the extracted MFCC features $MFCC^{i}$ (*i* represents the timestep) are fed into a gated recurrent unit (GRU) for each timestep, and the final hidden state $h_{A}^{t}$ of the GRU is concatenated with the prosodic feature vector to generate the representation vector of audio. In the TRE, the tokens of the text transcripts are passed through a word embedding layer, and the embedded tokens $\left(token^{1},\ldots ,token^{t}\right)$ are also fed into a GRU to obtain the representation vector of text. The text representation vectors extracted by TRE and the audio representation vectors extracted by ARE are concatenated and used for bimodal emotion recognition.

We propose a two-phase hierarchical network to simultaneously fuse the audio, text, and gesture information. In the first phase, the uni-modal features are fed into the ARE, the TRE, and the self-attention enhanced spatial temporal graph convolutional network, respectively, to obtain the audio, text, and gesture representations. In the second phase, these representations are passed through the fully connected layers and then concatenated to pass to the output layer to predict emotion.

## 5. Experiment

### 5.1. Dataset

We usde the IEMOCAP database [6] in the experiment. The IEMOCAP database records audio and video data when two actors have a dialogue in hypothetical or scripted scenarios. Every utterance is annotated into 10 classes by three or four annotators, i.e., happy, sad, angry, surprised, afraid, disgusted, frustrated, excited, or other. However, for body movement, only motion capture data of the head and hands motions, instead of body skeleton data, is contained in the database, thus ignoring some parts that are also crucial for emotional expression, e.g., the spine, the shoulders, and the arms.

**Figure 7:**
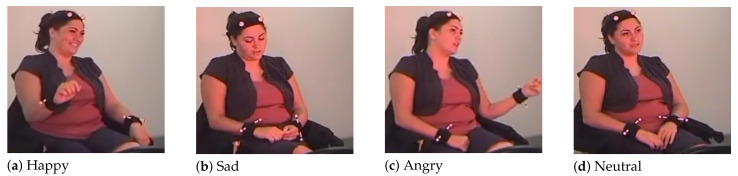
Illustration of examples of four emotions from IEMOCAP database [6].

To keep consistent with previous work, we merged the samples with excitement labels into the happiness subset and used four emotion classes, i.e., neutral, happy, sad, and angry. The dataset used in the experiment contained a total of 5492 samples in those four classes (1606 happy, 1081 sad, 1102 angry, and 1703 neutral). Some examples of the dataset are shown in Figure 7.

### 5.2. Feature Extraction and Experiment Setting

Following the work of [44], we used the OpenSMILE toolkit [45] to extract MFCC features and prosodic features and to initialize the tokens with the pretrained 300-dimensional GloVe vector [46]. The samples were divided into a training set, a development set, and a test set, with a ratio of 8: 0.5: 1.5 in the training process. The cross-entropy loss was used as the loss function to back-propagation.

### 5.3. Results

The performance of the proposed models and baseline models is shown in Table 1. We list the unweighted average recall (UAR) and the weighted average recall (WAR) of the unimodal and multimodal models utilizing audio signals, textual content, and body skeleton data. Considering the unbalance of the samples, the unweighted average recall was used to evaluate the model by treating each category equally. Since the value of the unweighted average recall was easily affected by the rare category, the weighted average recall was also applied to measure the overall prediction performance, which was numerically equal to the accuracy of the model.

For skeletal movement, ResNet18 [47] and ST-GCN [10] were selected as baseline models. ResNet is a convolutional network that adds a residual mechanism to the traditional convolutional neural network, which has been widely used in many tasks. In the experiment, we apply ResNet18 and ST-GCN to the skeleton data and compare their performance with our proposed model. Our S-STGCN model achieved a UAR of 68.4% and a WAR of 68.4%, significantly outperforming other networks in the emotion recognition task, which shows that the proposed model, especially the self-attention enhanced graph convolutional layer, could effectively extract emotion-related features from the dynamic skeleton sequence. It shows that the self-attention part obtained more flexible representations and provided effective supplementary information for the spatial graph convolution with the predefined graph. The two-stream architecture that integrated the joints and bones brought remarkable improvement, which indicates that this method could extract the emotional information from the skeleton modality more effectively.

We also compared the trimodal SERN with the MDRE [44] that was performed as the basic model of the audio and text modalities. Similarly, we applied the UAR and the WAR to the models as performance metrics. The model using text, audio, and skeleton information exceeded the MDRE that does not use skeleton information by a UAR of 8.4% and a WAR of 8.6%. The results show that the new extracted body skeleton modality contained some emotional information that was not contained in the audio and text modalities, and the use of the skeleton data enhanced the emotion recognition of the model. Moreover, we further compared a multimodal model using 2s-S-STGCN to extract skeleton features with the multimodal model using single-stream S-STGCN. The result showed that the use of the two-stream method also brought the improvement of the performance in multimodal emotion recognition.

## 6. Ablation Study and Discussion

### 6.1. Effect of the Preprocessing

In the data preprocessing (See Section 3.2), we applied the low-pass filter for the estimated position data of each joint to reduce the impact of the high-frequency noise. The performance of the preprocessed and unpreprocessed data is listed in Table 2. The result shows that the noise in the estimation result was harmful for skeleton-based emotion recognition and that the preprocessing brings considerable improvement.

### 6.2. Gating Mechanism

In Section 4.1.2, we introduced the gating mechanism to adjust the weight of the graph convolutional part and the self-attention part. In order to confirm the effect of the gating mechanism, the coefficient *r* was set to constant 1 in the ablation experiment. Thus Equation (9) was transformed into:

(10) $$f_{out}=\frac{f_{g}+f_{a}}{2}.$$

The result is shown in Table 3. The model using the gating mechanism obtained better prediction results, which suggests that the gating mechanism could flexibly adjust the importance of the self-attention part and was beneficial to the improvement of performance.

**Figure 8:**
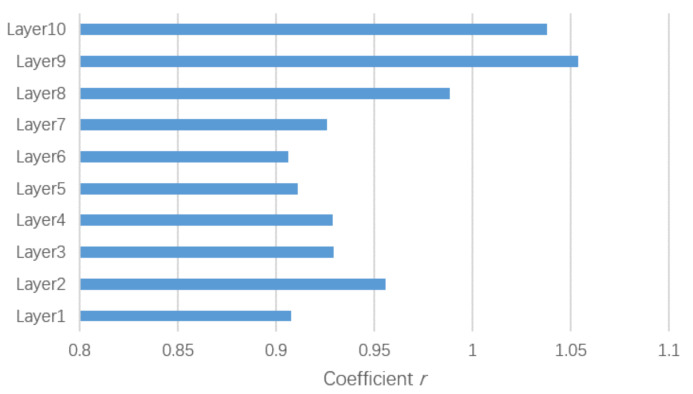
Visualization of the coefficient *r* of each layer.

We also visualized the coefficient *r* of each layer in Figure 8. The weights of the self-attention parts in the last several layers were higher than those in the other layers, and the weights in the last two layers were more than 1, which indicates that the features extracted by the self-attention part in the top layers were more informative than that of the bottom layers and that in last two layers the self-attention parts even were more important than the spatial graph convolutional part for the feature extraction.

### 6.3. Effect of the Bone Information

As introduced in Section 4.1.4, we fused the joint and the bone information to make the final prediction. Here we compared two fusion strategies: (i) The softmax scores of the two models were directly added as the final output. (ii) The scores were concatenated and then fed into a fully connected layer to obtain the result.

The experimental result is shown in Table 4. For single-input models, the performance of the joint model was slightly lower than that of the bone model. For the two-stream models with different fusion strategies, the trained model with the fully connected layer was better than the model using the summation strategy. Both of the two-stream models outperformed the single-input model, which shows the effectiveness of the two-stream architecture and the complementarity between the joint and bone information.

**Figure 9:**
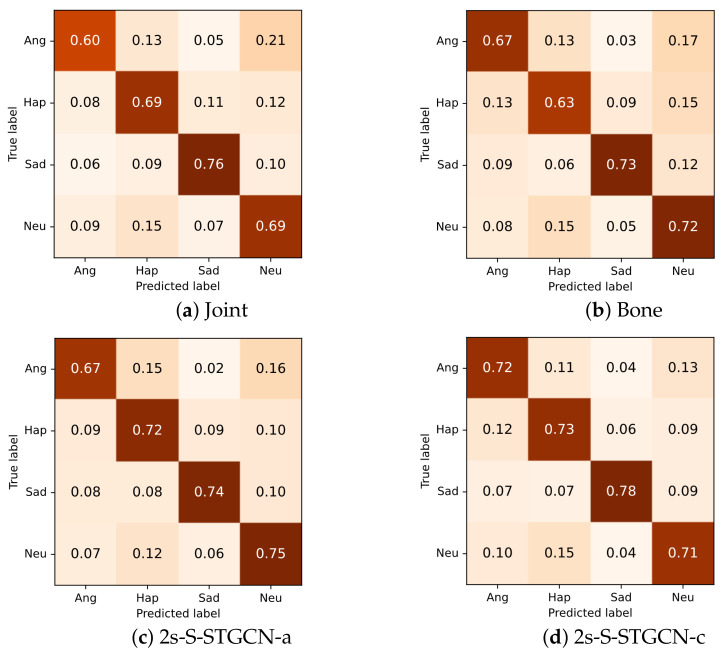
Confusion matrices for single-stream and two-stream models.

From the confusion matrix in Figure 9, it can be seen that the joint and bone modalities both performed best in the prediction of sad emotions. For joint information, the anger class was not well recognized, while for bone information, the samples of the happy class were often incorrectly recognized. For both of the single-stream models, other classes were frequently confused with the neutral class.

Overall, both of the two-stream models obtained better performance for most of the classes. The two-stream model using the summation of the scores also frequently incorrectly classified the samples of angry, happy, and sad classes as the neutral class. The other two-stream model that fed concatenation of the scores into the fully connected layer alleviated this problem to a certain extent and performed better on most of the categories.

### 6.4. Multimodal Analysis

**Figure 10:**
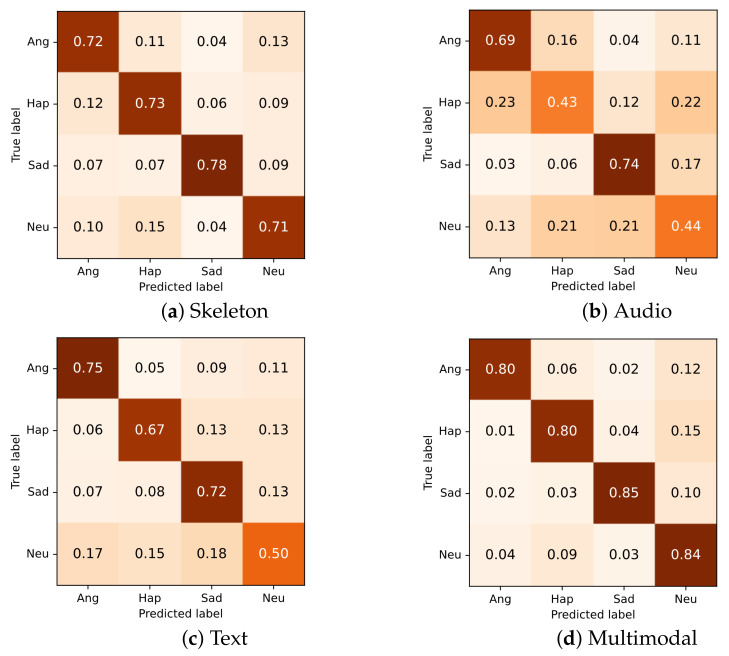
Confusion matrix for each modality. For skeleton modality, the two-stream self-attention enhanced spatial temporal graph convolutional network is used.

Different modalities showed different characteristics in the ability of emotion prediction. Figure 10 shows the confusion matrices of the unimodal and multimodal models. For skeleton modality (Figure 10a), the model showed the best performance in predicting the sad class. We speculate that this may be related to the posture of the spine and head. When people expressed their sadness, they tended to bend over and lower the head, which was obviously different from happy, angry, and neutral. As shown in (Figure 10b), for the audio model, anger and happiness were often incorrectly recognized as each other. For text modality (Figure 10c), although anger and happiness were correctly distinguished, angry, happy, and sad classes were frequently misclassified as the neutral class. The multimodal network that integrated the information from the three modalities (Figure 10d) achieved high and balanced performance for every class by leveraging the strengths of unimodal models and compensating for their weaknesses. From the error analysis, it can be seen that each modality had its unique strengths.

## 7. Conclusions

In this paper, we extracted body skeleton data using a pose estimation based approach from the videos of the IEMOCAP database. We proposed a novel two-stream self-attention enhanced spatial temporal graph convolutional network for emotion recognition based on the skeleton data. It models the body skeleton as a graph and constructs both static and flexible connections between the joints to update the representations. Through our experiment, we demonstrated that the performance of the proposed model is superior to that of the ST-GCN on emotion recognition tasks and that the two-stream architecture further improves the performance. Besides, we integrated information from audio signals, text transcripts, and skeletal movement, showing that our method outperforms the bimodal model, which indicates that the extracted skeleton data can provide important supplementary information for emotion detection. Since the multimodal fusion strategy that directly concatenates different modalities may not be optimal, we will focus on exploring more multimodal fusion strategies. We also plan to generate emotional gestures using the extracted skeleton data in our future work.

## Figures and Tables

**Table 1:** Comparison with baselines. We compare the performance of both the unimodal and multimodal models, for which A is the audio modality, T is the text transcription, and S is the skeleton data. SERN-2s is a skeleton enhanced emotion recognition network whose S-STGCN is replaced with 2s-S-STGCN.

Model	Modality	UAR(%)	WAR(%)
ARE [44]	A	59.7	57.1
TRE [44]	T	65.9	64.5
ResNet18 [47]	S	55.3	56.9
ST-GCN [10]	S	63.3	63.7
S-STGCN	S	68.4	68.4
2s-S-STGCN	S	**73.1**	**72.5**
MDRE [44]	A + T	72.0	71.4
SERN	A + T + S	80.4	80.0
SERN-2s	A + T + S	**82.2**	**82.3**

**Table 2:** Comparison between preprocessed and unpreprocessed data. The unpreprocessed noisy data and the preprocessed data are fed into S-STGCN respectively to compare their performance.

Data	UAR(%)	WAR(%)
Unpreprocessed	66.5	66.4
Preprocessed	68.4	68.4

**Table 3:** Comparison for the use of the gating mechanism.

Model	UAR(%)	WAR(%)
S-STGCN w/o G	67.7	67.3
S-STGCN	68.4	68.4

**Table 4:** Performances of different inputs and fusion strategies. J represents the joint information. B represents the bone information. 2s-S-STGCN-a is the two-stream model using the summation fusion strategy. 2s-S-STGCN-c is the two-stream model using the fully connected layer.

Model	Input	UAR(%)	WAR(%)
S-STGCN	J	68.4	68.4
S-STGCN	B	68.8	68.9
2s-S-STGCN-a	J&B	71.9	72.3
2s-S-STGCN-c	J&B	**73.1**	**72.5**
